# IPT-DCD: Interpolation Predictor for Teleoperation Under Dynamic Communication Delay Using Deep Learning Approach

**DOI:** 10.3390/s25134118

**Published:** 2025-07-01

**Authors:** Hwanhee Kang, Eugene Kim, Myeonghwan Hwang, Jaeguk Byeon, Jonghyeok An, Hyunrok Cha

**Affiliations:** 1 Robot Engineering, Korea National University of Science and Technology, 217 Gajeong-ro, Yuseong-gu, Daejeon 34113, Republic of Korea; hwan@kitech.re.kr (H.K.); wornr7390@kitech.re.kr (J.B.); ajh5265@kitech.re.kr (J.A.); 2 Purpose-Based Mobility Group, Seonam Division, Korea Institute of Industrial Technology, 6 Cheomdangwagi-ro 208beon-gil, Buk-gu, Gwangju 61012, Republic of Korea; egkim@kitech.re.kr (E.K.); han9215@kitech.re.kr (M.H.)

**Keywords:** artificial intelligence, remote operations and control, model-free prediction, delay compensation, LSTM, teleoperation

## Abstract

Teleoperation systems experience degraded control stability and safety due to dynamic communication delays. This study proposes an Interpolation Predictor for Teleoperation under Dynamic Communication Delay (IPT-DCD), a predictor that reconstructs asynchronously received control commands via interpolation and predicts future commands using an encoder–decoder LSTM architecture. To restore the temporal consistency of delayed signals, a signal preprocessing technique called the Backward Shifting and Interpolation (BSI) was applied, enabling the transformation of received data into an undelayed and uniformly sampled format. As a result, the proposed model was capable of generating real-time steering command outputs through a many-to-many time series structure. Furthermore, to evaluate its effectiveness, IPT-DCD was experimentally compared with a baseline model, a Predictor for Teleoperation under Dynamic Communication Delay (PT-DCD). The results reveal that IPT-DCD exhibits significantly greater robustness to large communication delay outliers than the baseline, highlighting its effectiveness in dynamic and unstable teleoperation environments.

## 1. Introduction

Teleoperation has been employed across various domains including on-road vehicles [1], military operations [2], industrial automation [3], and medical surgery [4]. Recent studies have focused on improving teleoperation performance under varying communication conditions, frequently incorporating human-in-the-loop approaches that enhance overall control performance and safety, as summarized in Table 1. The variability in operational environments and network quality is largely influenced by the type of network connecting operators and devices, as well as the specific teleoperation application. One of the most critical characteristics of teleoperation systems is the presence of communication delays, which can lead to a discrepancy between the operator’s intent and the actual movement of the controlled device [5,6,7]. These communication delays can pose significant risks to system performance and operator safety. To mitigate the adverse effects of communication delays, recent research has focused on developing delay compensation techniques, both through physical modeling approaches and model-free methodologies.

**Figure 1:**
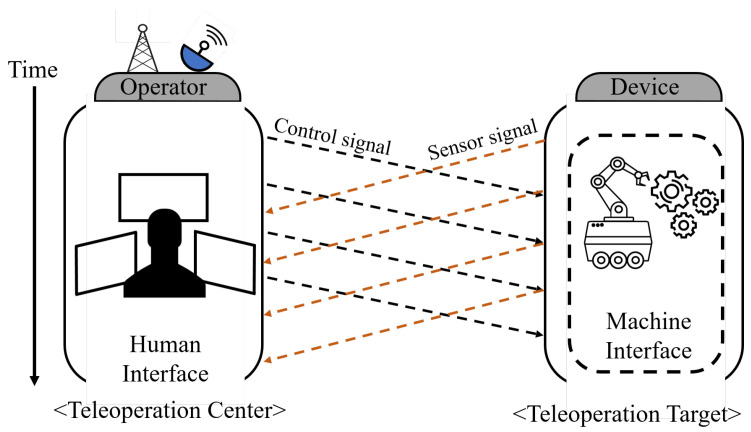
Schematic diagram of communication delay and its classification: control signal and sensor signal delay.

As depicted in Figure 1, communication delays can generally be classified into control delay, which emerges during the transmission of a remote control signal, and sensor delay, which occurs during the reception of a sensor signal [15]. Numerous techniques have been proposed to ensure the stability and performance of teleoperation systems by mitigating both control and sensor delays, with some methods achieving success through model predictive control (MPC) [16]. Subsequently, more generalized predictive models, such as the Smith predictor, gained prominence due to the limitations of strict MPC-based predictors that require highly accurate plant models [17]. Early work improved sliding-mode control, $H_{\infty }$ design, and networked control by introducing probabilistic analyses and robust filtering [18,19,20]. Additionally, while the model-based approach can predict control commands and sensor values based on physical movement, it may introduce discrepancies due to accumulated model errors [15]. Therefore, some studies have adopted model-free predictors and pursued more robust passivity-based approaches [21]. Variable-transformation (wave-scattering) methods later proved robust to nonlinear dynamics and time-varying delays precisely because they bypass explicit plant modelling [22,23]. In addition, several studies have applied model-free predictors to passivity control and energy balance monitoring, which have contributed greatly to improving the stability of teleoperation as a bilateral controller [24,25,26,27]. Meanwhile, the model-free approach often exhibits lower performance than the model-based approach; researchers have therefore proposed various improvements [11,15,21,28,29]. Since then, these model-free predictors have been supplemented and modified to adapt to delay fluctuation caused by uncertain network conditions [30]. Nevertheless, some methods are still criticised for residual high-frequency oscillations [31]. Table 2 summarizes these approaches.

**Figure 2:**
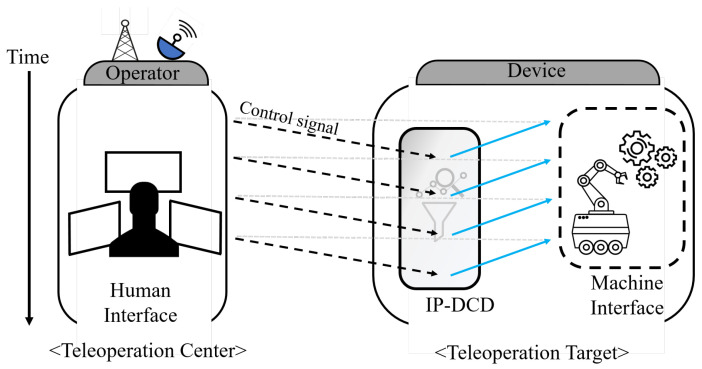
Concept of the IPT-DCD (Interpolation Predictor for Teleoperation under Dynamic Communication Delay) for compensation of communication delay.

Another important trend is the rapid development of AI. Deep learning-based predictors utilizing RNN (Recurrent Neural Network)-based time series forecasting (TSF) are now actively studied [39]. Sophisticated models using LSTM and GRU, which represent RNNs, can be interpreted as data-driven model-free predictors; unlike other model-free approaches, they do not require strict dynamics. Recently, predictors utilizing techniques such as LSTM, a type of RNN, and high-degree polynomial linear regression for unavoidable transmission delays have been reported, and demonstrations for real-time operation have been conducted [40]. In addition, studies have explored LSTM-based predictors that compensate for input delays using LSTM-integrated MPC in nonlinear time-delay systems [41]. LSTM or GRU offers many strengths in time series processing, and recently, Attention-based models such as the Transformer, famous for GPT, have also been widely studied in the TSF field [42]. Although incorporating Attention mechanisms could enhance prediction accuracy by selectively emphasizing critical input features, their application introduces additional computational overhead and increased memory requirements. Specifically, Attention mechanisms involve frequent computation of weight matrices, thereby elevating computational complexity and inference latency, which are critical drawbacks for real-time teleoperation tasks performed in resource-constrained edge computing environments. Furthermore, Attention mechanisms typically offer meaningful benefits primarily when modeling longer sequences or large-scale datasets. In contrast, LSTM and GRU are more computationally efficient for short-term predictions (∼few seconds), aligning precisely with the goals of this research. Therefore, this study applied supplementary techniques as a preprocessing step to enhance LSTM-based prediction under dynamic network conditions [43]. Between GRU and LSTM, GRU converges faster during training, but LSTM typically demonstrates superior accuracy for slightly longer sequence predictions. Considering these trade-offs and the requirements of this research, LSTM was selected for this study. To the best of the authors’ knowledge, most model-free predictors using neural networks estimate the next physical value over time, and there are few studies on predicting values after a specific communication delay τ. In particular, to effectively compensate for control delay, it is important to precisely measure the one-way communication delay rather than round-trip time (RTT). Such compensation should be based on this one-way delay measurement, especially when dealing with realistic and time-varying delays instead of constant delays. For this reason, we propose the IPT-DCD (Interpolation Predictor for Teleoperation under Dynamic Communication Delay), as shown in Figure 2.

### Contribution

- This paper proposes IPT-DCD, a method designed to compensate for communication delays by predicting delayed signals with an interpolation method.
- The proposed IPT-DCD is formulated as a data-driven, model-free framework, which enables flexible adaptation to various systems through training on samples collected from the target operating environment.
  - IPT-DCD enhances the previously introduced PT-DCD by integrating the BSI (Backward Shifting and Interpolation) technique, thereby achieving improved robustness in prediction performance.
- We conducted a series of experiments across diverse scenarios by varying the proportion of delay outliers, showing that IPT-DCD can maintain the performance even in unstable conditions.
- The proposed approach exhibits robustness against communication delays for a wide range of teleoperation applications, particularly offering reliable dynamic delay outlier compensation capabilities.

## 2. Materials and Methods

In this study, we utilized a Recurrent Neural Network (RNN)-based predictor to overcome the limitations in existing model-free prediction approaches, which often fail to capture the full extent of system dynamics [44]. Previous research has demonstrated the efficacy of RNN-based architectures, such as Long Short-Term Memory (LSTM) and Gated Recurrent Units (GRUs), in learning complex nonlinear and temporal dynamics [45,46]. These architectures are now considered well suited for modeling behaviors in time-dependent systems, as they exhibit superior performance in capturing regional patterns within dynamic environments. However, RNN-based predictors often struggle to accurately predict the target in the presence of complex dynamic systems and coexisting communication delays. Therefore, this study focuses on improving a novel LSTM-based predictor framework by considering communication delay constraints.

### 2.1. Problem Definition

In practice, communication delays can be categorized into processing, queueing, transmission, and propagation delays [47]. However, from the perspective of operator-device system architecture, these delays can be roughly classified into two main types, control delay $\tau_{1}\left(t\right)$, and sensor delay $\tau_{2}\left(t\right)$:

(1) $$\tau_{\mathrm{RTT}}\left(t\right)=\tau_{1}\left(t\right)+\tau_{2}\left(t\right),$$

where $\tau_{\mathrm{RTT}}\left(t\right)$ denotes a round trip communication delay at time *t*.

**Figure 3:**
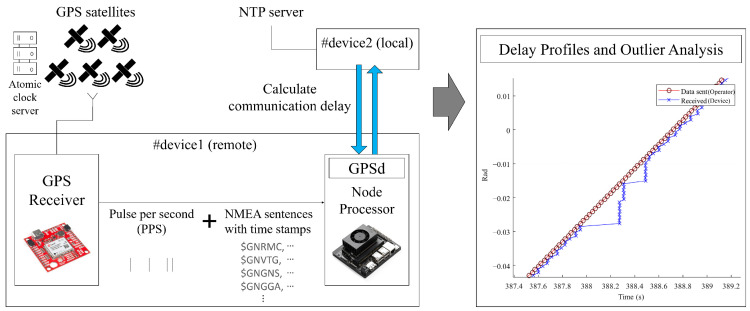
Construction of delay profiles and outlier analysis using time synchronization with GPS and an NTP server.

A general closed-loop system over a network where two distant subsystems exchange information with each communication delay can be expressed as follows:

(2) $$\begin{array}{ccc} {\hat{x}}_{i}\left(t\right) & = & g_{i}(x_{i}\left(t-\tau_{i}\left(t\right)\right), \end{array}$$

(3) $$\begin{array}{ccc} h_{i}\left(x_{i}\left(t\right)\right) & = & x_{i}\left(t-\tau_{i}\left(t\right)\right), \end{array}$$

where (i={1,2}) represents each subsystem, $x_{i}\left(t\right)$, ${\hat{x}}_{i}\left(t\right)$ denotes the actual and predicted state space vectors, and $g_{i}\left(\cdot \right)$ and $h_{i}\left(\cdot \right)$ are nonlinear functions for prediction and observation respectively, while $\tau_{i}\left(t\right)$ represents communication delay for each subsystem. To independently measure control and sensor delays, high-precision GPS receivers with PPS (Pulse Per Second) functionality can be used for accurate time synchronization. As shown in Figure 3, the GPS provides PPS signals and time-stamped NMEA messages to connected devices. The local device uses an NTP server for clock sync, while the remote device uses GPS timestamps. Consequently, the one-way communication delay can be precisely measured. By accumulating communication logs, a delay profile is generated for analyzing delay distributions and detecting outliers under varying network conditions [6]. According to the literature, many stochastic approaches were introduced to model the communication delays (i.e., Heavy-tailed Gaussian, stochastic Gaussian mixture, generalized extreme value distribution, etc.) [6,11,48]. In this framework, we assume the communication delay follows the stochastic Gaussian mixture model to represent passive and outlier communication delays probabilistically [6]. Therefore, the control delay $\tau_{1}\left(t\right)$ can be written as

(4) $$\tau_{1}\left(t\right)∼\left(1-\rho \right)N\left(\mu_{p},s_{p}^{2}\right)+\rho N\left(\mu_{o},s_{o}^{2}\right),\;0\leq \rho \leq 1,$$

where ρ is a contamination ratio of the communication delay outlier, $\mu_{p}$, and $s_{p}$ are the mean and standard deviation of the passive delay distribution, $\mu_{o}$, and $s_{o}$ are the mean and standard deviation of the outlier delay distribution.

Finally, we set our objective to minimize the error between the predicted control signal and the state space vector at time *t*, which can be expressed as

(5) $$\epsilon \left(t\right)=x\left(t\right)-f_{i}\left(x_{i}\left(t-\tau_{i}\left(t\right)\right)\right),$$

where ϵ(t) denotes the prediction error vector between x(t) and $\hat{x}\left(t\right)$. In summary, Table 3 shows notations used in problem definition.

### 2.2. Long Short-Term Memory Network

**Figure 4:**
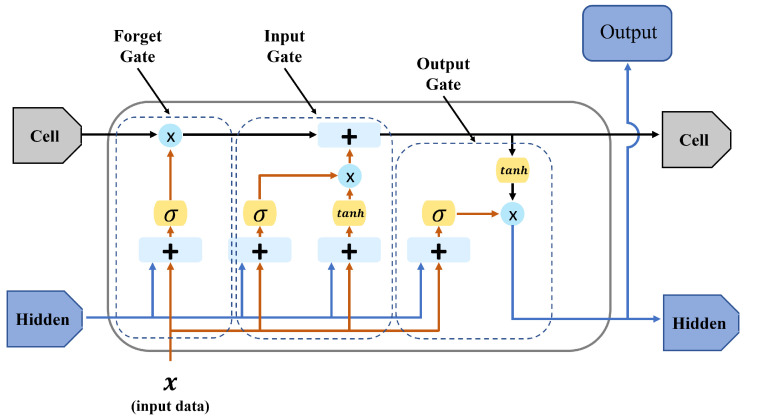
Architecture of LSTM network cell. The LSTM cell consists of one cell state and three primary gates: input gate, forget gate, and output gate.

LSTM networks are sophisticated recurrent neural networks that excel at processing data where input order is important. LSTM Structure: Each LSTM unit (or cell) contains a cell state and three primary gates: the input gate, forget gate, and output gate. Figure 4 shows the structure of the single LSTM cell. Firstly, a cell state term is designed to either directly propagate significant information (long-term information) from previous LSTM cells or pass the information based on the network’s requirements:

(6) $$c_{t}=f_{t}*c_{t-1}+i_{t}*{\tilde{c}}_{t},$$

where $c_{t}$ is the current content vector using both the forget and input gates, $f_{t}$ is a forget gate term, and $i_{t}$ is an input gate term at time *t* which controls how much of the new candidate information ${\tilde{c}}_{t}$ should be added to the cell state.

Next is the forget gate, which is responsible for regulating the amount of information to be discarded from the previous cell state. By applying a forgetting mechanism, it selectively filters out irrelevant or outdated information, ensuring that the cell state retains only pertinent data:

(7) $$f_{t}=\sigma \left(W_{f_{h}}\left[h_{t-1}\right],W_{f_{x}}\left[x_{t}\right],b_{f}\right),$$

where $f_{t}$ is the forget gate at time *t*, σ is a non-linear function, such as ReLU or the hyperbolic tangent function, $W_{f}$ is a weight matrix associated with forget gate, $h_{t-1}$ is a previous hidden state, $x=(x_{1},x_{2},\ldots ,x_{t})$ is the input vector, and $b_{f}$ denotes bias vector of the forget gate.

The input gate processes the current input alongside the previous hidden state to determine how much the new information should be incorporated into the cell state. The input gate mechanism facilitates the controlled integration of new input to the LSTM network’s memory:

(8) $$i_{t}=\sigma \left(W_{i_{h}}\left[h_{t-1}\right],W_{i_{x}}\left[x_{t}\right],b_{i}\right),$$

where $i_{t}$ is the input gate term, $W_{i}$ is a weight matrix associated with the input gate, and $b_{i}$ is the bias vector of the input gate.

Finally, the output gate rules the proportion of overall information from the cell state that is connected to the output of the LSTM unit. The output gate modulates the extent to which the current cell state influences the subsequent hidden state and network output:

(9) $$o_{t}=\sigma \left(W_{o_{h}}\left[h_{t-1}\right],W_{o_{x}}\left[x_{t}\right],b_{o}\right),$$

where $o_{t}$ denotes the final output vector, $W_{o}$ is a weight matrix associated with the output gate, and $b_{o}$ is the bias vector of the output gate. In addition, the hidden state cell, which represents the intermediate output emitted from the previous LSTM cell contains information about the prior hidden state of the network:

(10) $$h_{t}=o_{t}*\tan h\left(c_{t}\right),$$

where $h_{t}$ is the hidden state cell at time *t*. In summary, notations regarding the LSTM network are shown in Table 4.

### 2.3. Neural Network-Based Prediction Method

In this subsection, the PT-DCD method previously proposed in [49] and the IPT-DCD method are introduced. Both methods utilize an LSTM-based encoder–decoder architecture to predict control signals that are compensated for communication delay. The key distinction of IPT-DCD lies in the application of the BSI (Backward Shifting and Interpolation) process, which enhances robustness against outlier noise compared to PT-DCD.

#### 2.3.1. PT-DCD (Baseline Method)

**Figure 5:**
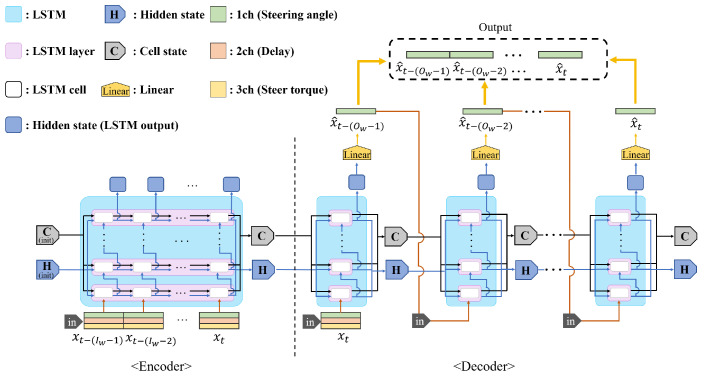
Overall structure of PT-DCD (Predictor for Teleoperation under Dynamic Communication Delay), which predicts the next control signal based on the past trends in the received control signals.

**Figure 6:**
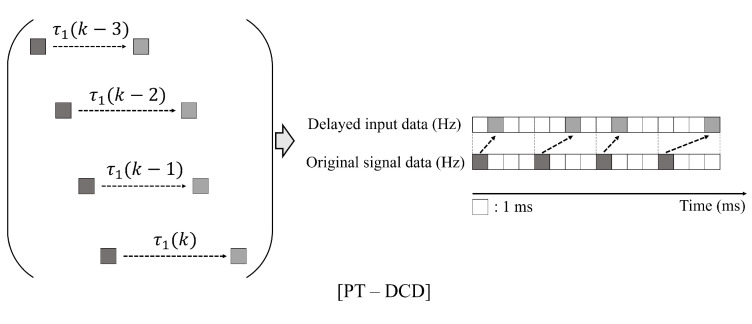
Schematic representation of the original control signal. Its delayed counterpart is also presented. Due to independent communication delays, the delayed control signals do not exhibit uniform time intervals compared to the original signal.

Figure 5 shows a Seq-2-Seq PT-DCD structure composed of LSTMs. The PT-DCD (Predictor for Teleoperation under Dynamic Communication Delay) method is a basic form of predictor that predicts the next control signal based on the existing LSTM. The input of the PT-DCD requires discrete input vector elements:

(11) $$g_{p}\left({\tilde{X}}_{k}|\Theta \right)={\hat{Y}}_{k},$$

where $g_{p}$ is a non-linear function used in the PT-DCD LSTM network for prediction. The input and output are ${\tilde{X}}_{k}$, ${\hat{Y}}_{k}$ which can be rewritten as

(12) $$\begin{array}{ccc} {\tilde{X}}_{k} & = & {\left[{\tilde{x}}_{k},{\tilde{x}}_{k-1},\ldots ,{\tilde{x}}_{k-w_{i}+1}\right]}^{T}, \end{array}$$

(13) $$\begin{array}{ccc} {\hat{Y}}_{k} & = & {\left[{\hat{x}}_{k},{\hat{x}}_{k-1},\ldots ,{\hat{x}}_{k-w_{o}+1}\right]}^{T}, \end{array}$$

(14) $$\begin{array}{ccc} {\tilde{x}}_{k} & = & x(k-\tau_{1}\left(k\right)), \end{array}$$

where $w_{i}$, $w_{o}$ denote a predefined input and output window size of the dataset. It should be noted that $\tilde{x}$ represents the measured state space vector as shown in Figure 6.

**Figure 7:**
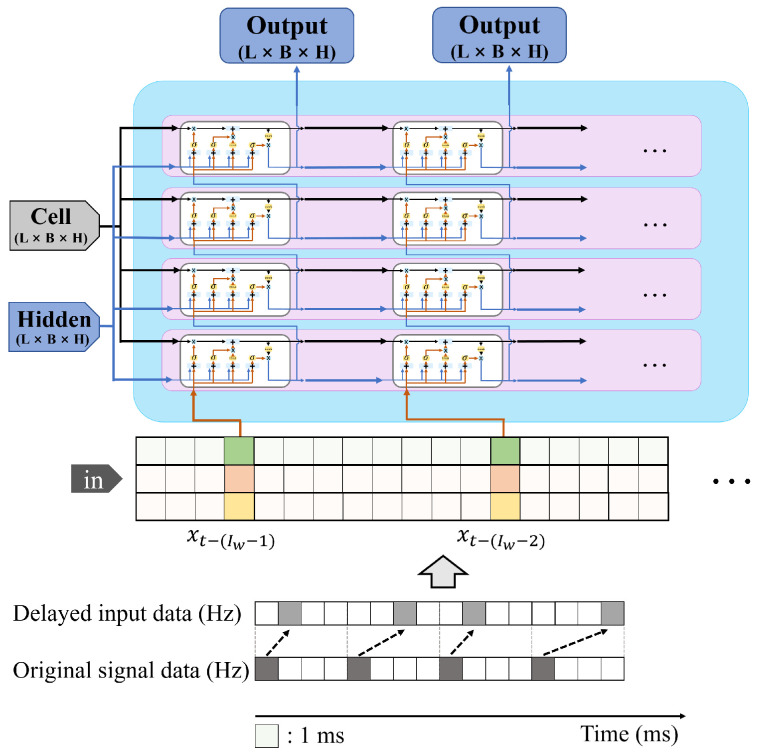
Graphical representation of PT-DCD method which is based on a discrete prediction model and only considers the amount of control delay at time *k*. Note that the delayed control signal, the amount of delay, and the delayed steering torque are used as inputs without any pre-processing.

More precisely, since we focus on compensating for the control delay, the input vector ${\tilde{x}}_{k}$ can be written as

(15) $$\begin{array}{ccc} {\tilde{x}}_{k} & = & {\left[\tilde{\theta_{k}},\tau_{1}\left(k\right)\right]}^{T}, \end{array}$$

(16) $$\begin{array}{ccc} \tilde{\theta_{k}} & = & \theta (k-\tau_{1}\left(k\right)), \end{array}$$

where ${\tilde{\theta }}_{k}$ is the observed control signal and $\tau_{1}\left(k\right)$ is the control delay at discrete time *k*. Therefore, based on the PT-DCD method, (5) can be rewritten to represent prediction error:

(17) $$\epsilon_{p}\left(k\right):=x\left(k\right)-g_{p}\left({\tilde{X}}_{k}\right){|}_{n},$$

where $g_{p}\left({\tilde{X}}_{k}\right){|}_{n}$ denotes the *n*th element vector of PT-DCD output. As shown in Figure 7, the PT-DCD model is based on a discrete prediction model which only accounts for the control delay at time *k* but does not consider sample uniformity or balance. In summary, notations used for explaining PT-DCD are presented in Table 5.

#### 2.3.2. IPT-DCD (Proposed Method)

To enhance the accuracy of predicting the original non-delayed state space vector, IPT-DCD (Interpolation Predictor for Teleoperation under Dynamic Communication Delay) is further developed to overcome the limitations of PT-DCD. Accordingly, the predictor of IPT-DCD can be described as

(18) $$g_{\mathrm{ip}}\left({\tilde{X}}_{t}|\Theta \right)={\hat{Y}}_{t},$$

where ${\tilde{X}}_{t}$ is a state space matrix at time *t* based on observation, ${\hat{Y}}_{t}$ is an output matrix of $g_{\mathrm{ip}}$ which is the non-linear IPT-DCD LSTM function, and Θ represents the network parameters.

Firstly, the uniformly observed state-space matrix ${\tilde{X}}_{t}$ is the set of state-space vectors for a certain time interval:

(19) $$\begin{array}{ccc} {\tilde{X}}_{t} & = & {\left[{\tilde{x}}_{t},{\tilde{x}}_{t-T_{p}},\ldots ,{\tilde{x}}_{t-\left(w_{i}-1\right)T_{p}}\right]}^{T}, \end{array}$$

(20) $$\begin{array}{ccc} {\hat{Y}}_{t} & = & {\left[{\hat{x}}_{t},{\hat{x}}_{t-T_{p}},\ldots ,{\hat{x}}_{t-\left(w_{o}-1\right)T_{p}}\right]}^{T}, \end{array}$$

(21) $$\begin{array}{ccc} {\tilde{x}}_{t} & = & x(t-\tau_{1}\left(t\right)), \end{array}$$

where $f_{p}$ is the interpolation sampling frequency of the original sample and the period between each sample is $T_{p}=\frac{1}{f_{p}}$. Accordingly, (15) can be rewritten as

(22) $$\begin{array}{ccc} {\tilde{x}}_{t} & = & {\left[\tilde{\theta_{t}}\tau_{1}\left(t\right)\right]}^{T}, \end{array}$$

(23) $$\begin{array}{ccc} \tilde{\theta_{t}} & = & \theta (t-\tau_{1}\left(t\right)). \end{array}$$

However, what can practically be observed from the delayed control samples is a set of non-uniformly obtained samples:

(24) $${\tilde{X}}_{k}={\left[{\tilde{x}}_{k},{\tilde{x}}_{k-1},\ldots ,{\tilde{x}}_{k-w_{i}+1}\right]}^{T},$$

where ${\tilde{X}}_{k}$ is the observed state space model and the elements corresponding to ${\tilde{X}}_{k}$ can be written as

(25) $$\begin{array}{ccc} {\tilde{x}}_{k} & = & {\left[\tilde{\theta_{k}},\tau_{1}\left(k\right)\right]}^{T}, \end{array}$$

(26) $$\begin{array}{ccc} \tilde{\theta_{k}} & = & \theta (k-\tau_{1}\left(k\right)). \end{array}$$

**Figure 8:**
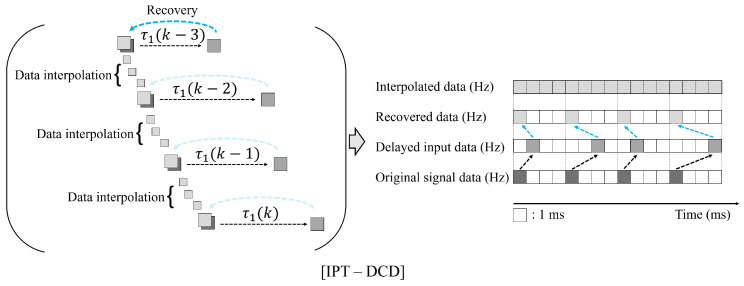
Schematic representation of the original control signal and its restored control signal after interpolation. The IPT-DCD (Interpolation Predictor for Teleoperation under Dynamic Communication Delay) initially restores the delayed control signal based on the measured communication delay and subsequently performs interpolation between the samples as a preprocessing step.

As shown in Figure 8, IPT-DCD performs the Backward Shifting and Interpolation (BSI), a preprocessing step that realigns delayed samples to their original timestamps by subtracting the corresponding delay from their received times and subsequently interpolates signal values between these adjusted samples:

(27) $$\begin{array}{cccc} p(\Delta t|{\tilde{\theta }}_{k:1}) & =h\left(\theta_{k-1}\right)+\frac{h\left(\theta_{k}\right)-h\left(\theta_{k-1}\right)}{T_{s}}\Delta t, & \; & (\mathrm{only}\;\mathrm{when}\;\Delta t\leq T_{s}), \end{array}$$

where $T_{s}$ is the original sampling frequency of the control signal and Δt is the desired time interval based on a predefined interpolation rate (i.e., $T_{p}$). As $T_{p}$ decreases, prediction resolution improves, enhancing the accuracy of future steering command estimation. The IPT-DCD predicts $w_{o}$ future control steps and selects the output corresponding to the measured delay $\tau_{1}\left(t\right)$. If $\tau_{1}\left(t\right)$ lies between prediction steps, the nearest predicted value is used. Smaller $T_{p}$ reduces substitution error, improving prediction accuracy. However, it shortens the temporal coverage of the fixed input window, limiting the time-series context the predictor can utilize. To compensate, a larger input window is required, increasing computational cost. Thus, $T_{p}$ must be chosen to balance prediction accuracy and computational efficiency. Accordingly, we let *P* be the process that uses (27), and the final input state space matrix can be written as

(28) $$P\left({\tilde{X}}_{k}\right)=\left[{\tilde{x}}_{t},{\tilde{x}}_{t-T_{p}},\ldots ,{\tilde{x}}_{t-\left(w_{i}-1\right)T_{p}}\right].$$

Next, the mean squared error is used to optimize and obtain IPT-DCD network parameters:

(29) $$\begin{array}{c} L\left(\Theta \right)=\frac{1}{N}\sum_{i=1}^{N}\left|\theta_{t_{i}}-{\hat{\theta }}_{t_{i}}\right|, \end{array}$$

(30) $$\begin{array}{c} \Theta^{*}=\min L\left(\Theta \right), \end{array}$$

where L is the loss function and *N* is the total number of data samples.

**Figure 9:**
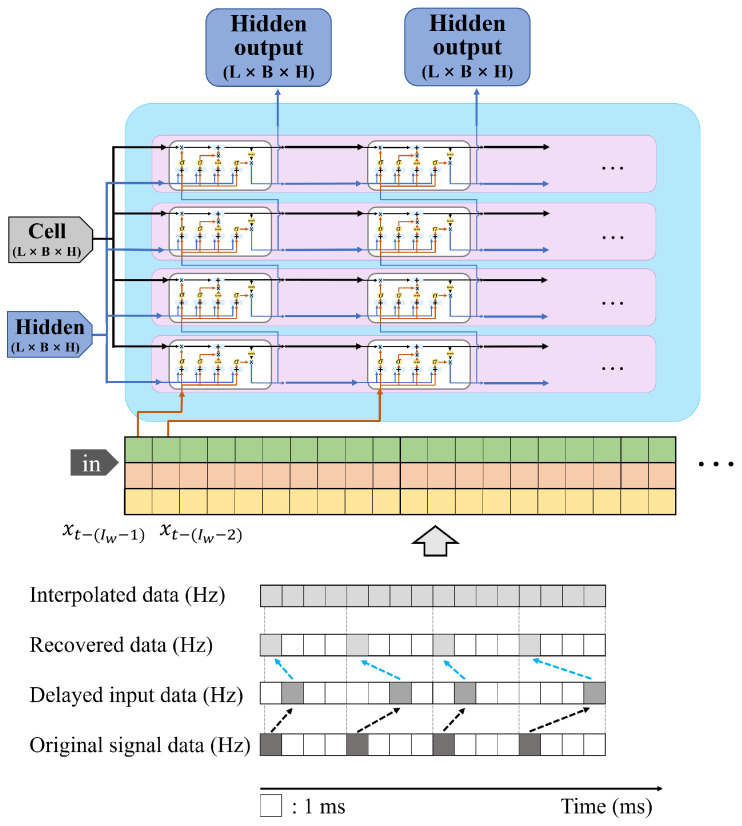
Graphical expression of the IPT-DCD (Interpolation Predictor for Teleoperation under Dynamic Communication Delay), which includes BSI preprocessing.

Figure 9 illustrates the BSI process applied to each input signal to construct the input datasets.

In summary, notations used for describing IPT-DCD are shown in Table 6.

## 3. Experiments

This section describes the experimental setup and validation process for evaluating the effectiveness and suitability of the proposed method, IPT-DCD.

### 3.1. General Setups for Network Training

For training and validation, artificial communication delays were generated using (4) where $\mu_{p},s_{p},\mu_{o}$, and $s_{o}$ were set to 0.03 s, 0.01 s, 0.113 s, and 0.05 s, respectively. The contamination ratio (ρ) was set to 0.1 according to reference [6]. For the training dataset, steering angle and steering torque values were extracted from dataset [50] provided by Comma.ai. Then, artificially generated communication delays were applied to these values to create delayed steering commands.

### 3.2. Training of the IPT-DCD

IPT-DCD was trained using sequences of steering angles, communication delays, and steering torques as input data, which represent delayed steering commands. In (28), the input window size $w_{i}$ and the sampling period $T_{p}$ were set to 20 and 0.1 s, respectively. Table 7 shows the parameters used for IPT-DCD training. The output window size $w_{o}$ was 10, which means that IPT-DCD simultaneously produces future values in the range of 0–100 ms and uses the index corresponding to the current $\tau_{1}$ (measured one-way communication delay at time *t*) as the predicted real-time steering command.

**Figure 10:**
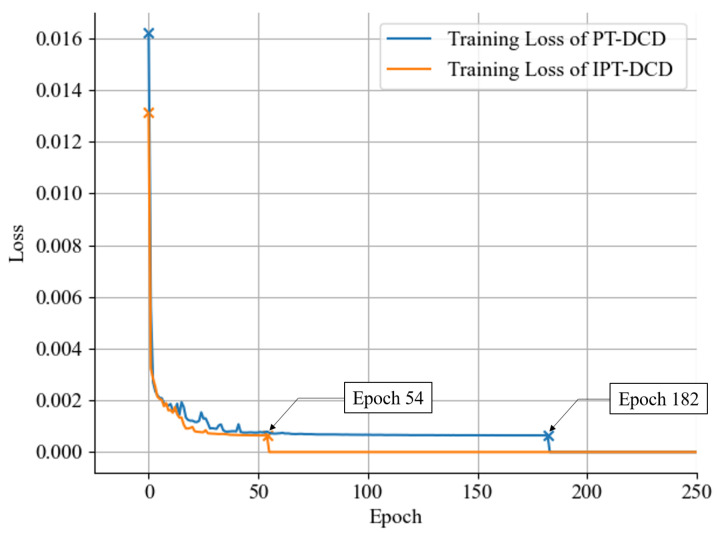
Learning curve graph of the PT-DCD and the IPT-DCD.

Using the early stopping technique, IPT-DCD was trained for 182 epochs, while PT-DCD was trained for 54 epochs. As shown in Figure 10, the training loss of IPT-DCD decreased faster compared to PT-DCD, demonstrating faster convergence. Computational performances and versions of AI-related software for training are shown in Table 8.

### 3.3. Validation of the IPT-DCD

To verify the robustness of IPT-DCD, validation was performed under both the trained condition (ρ=0.1) and additional untrained conditions with higher contamination ratios (ρ=0.3 and ρ=0.5). Although the real teleoperation environment typically corresponds to ρ=0.1, the condition with ρ=0.3 was selected to simulate scenarios with unstable communication. The scenario with ρ=0.5, representing an extreme communication condition where outliers account for half of the total communication delay, is unlikely in real-world teleoperation environments. However, this extreme case was included to evaluate IPT-DCD’s performance under highly challenging conditions. Additionally, predictions were performed under an ideal scenario without any outliers (ρ=0) for further comparison. For the Monte Carlo analysis, IPT-DCD performed 100 prediction trials on validation samples, varying only the communication delay parameter. The results were then compared with PT-DCD predictions.

## 4. Results

In this section, the results of PT-DCD and IPT-DCD validation are shown.

### 4.1. Performance of the PT-DCD

Table 9 shows RMSE values with and without PT-DCD according to ρ. RMSE decreased by 39.90% using PT-DCD when ρ=0, and then the reduction percentage was becoming smaller with increasing ρ.

### 4.2. Performance of the IPT-DCD

Table 10 compares the RMSE of delayed steering signals with those using IPT-DCD. With IPT-DCD, the smallest RMSE reduction was observed at ρ=0, and the effect became larger as ρ increased. This demonstrates a trend that is in direct contrast to PT-DCD, where the RMSE reduction effect diminished as ρ increased.

### 4.3. Intersection of Prediction Performance

**Figure 11:**
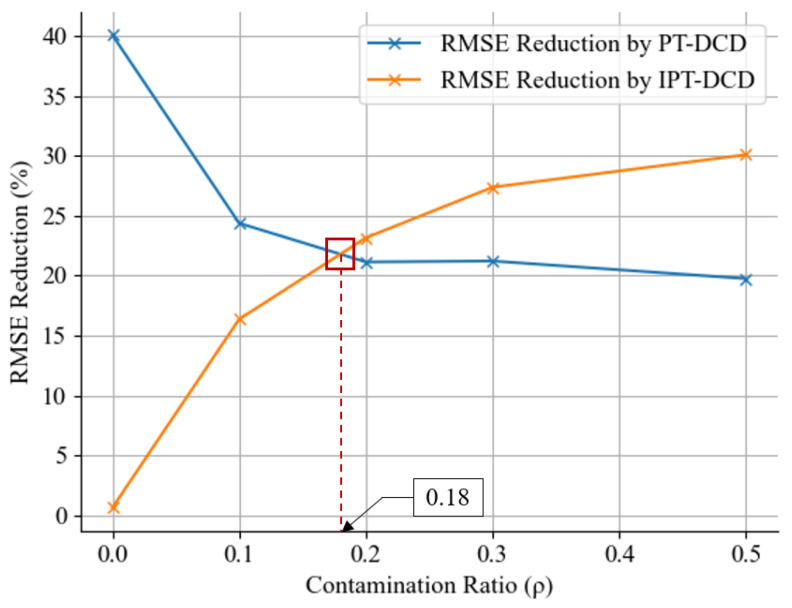
RMSE reduction curves for the PT-DCD and the IPT-DCD intersect at approximately ρ=0.18.

When comparing the results of the two methods, PT-DCD exhibits a decreasing RMSE reduction effect as ρ increases, whereas IPT-DCD shows an increasing effect. As depicted in Figure 11, the two graphs are estimated to intersect at approximately ρ=0.18.

### 4.4. Statistical Analysis

This section presents the statistical analysis conducted to verify whether the performances of PT-DCD and IPT-DCD differ significantly.

#### 4.4.1. Normality Test—Shapiro–Wilk Test

Firstly, we performed a normality test using the Shapiro–Wilk method on the RMSE data grouped by predictors (PT-DCD and IPT-DCD) and the contamination ratio ρ. According to the Shapiro–Wilk test, the normality assumption is rejected if a group’s *p*-value is smaller than 0.05. Twelve groups in total were tested, comprising delayed steering angles and predicted steering angles by PT-DCD and IPT-DCD at four different values of ρ. The results of the normality test are summarized in Table 11. Ten out of the twelve groups satisfied the normality assumption, whereas two groups did not. Therefore, it was concluded that the data did not fully meet the assumption of normality required for comparative analysis.

#### 4.4.2. Nonparametric ANOVA—Kruskal–Wallis Test

Secondly, the Kruskal–Wallis test was conducted to determine whether there were statistically significant differences among the RMSE groups. Table 12 presents the results. The Kruskal–Wallis test is a nonparametric method suitable when the data do not meet the normality assumption. At each ρ, the RMSE values of the Delayed, PT-DCD, and IPT-DCD groups were compared. The results show that there were significant differences between the groups in all cases.

#### 4.4.3. Dunn’s Test

As a post hoc analysis following the Kruskal–Wallis test, Dunn’s test was performed to identify specific pairs of groups that differed significantly. As shown in Table 12, the *p*-values from all pairwise comparisons except between IPT-DCD and Delayed at ρ=0 were below 0.05, indicating that the median RMSE values significantly differed between all groups.

#### 4.4.4. Effect Size—Cohen’s d

Lastly, Cohen’s d values were calculated to quantitatively assess the magnitude of differences between the groups. Calculated Cohen’s d values for each group are shown in Table 13.

## 5. Discussion

### 5.1. Performance Comparison Between the PT-DCD and the IPT-DCD

The statistical analysis clearly showed a significant difference between IPT-DCD and PT-DCD, proving that the method applied to IPT-DCD is indeed a novel approach distinct from PT-DCD. By introducing the BSI process, IPT-DCD is able to perform predictions at uniform time intervals, effectively reducing the RMSE under all experimental conditions. Under the training condition of ρ=0.1, PT-DCD achieved a greater RMSE reduction (24.38%) compared to IPT-DCD (16.37%), indicating relatively lower performance by IPT-DCD in stable conditions. However, as ρ increases, the two methods exhibit opposite performance trends, demonstrating the distinct operating characteristics of each and reinforcing the novelty of the BSI-based approach.

### 5.2. Impact of Outliers on the Performance of the PT-DCD and the IPT-DCD

PT-DCD and IPT-DCD were evaluated under four different conditions, with ρ = 0, 0.1, 0.3, and 0.5 representing the proportion of outliers in communication delay. The case of ρ=0.5 corresponds to a relatively extreme communication environment, where outliers account for half of the total delay. Even under this condition, PT-DCD achieved an RMSE reduction of nearly 20%. Moreover, IPT-DCD achieved an RMSE reduction of approximately 30%, demonstrating a greater improvement than PT-DCD.

### 5.3. Efficient Application of the PT-DCD and the IPT-DCD

An interesting observation is that IPT-DCD does not consistently outperform PT-DCD; rather, it demonstrates an opposite trend depending on the variation in the contamination ratio (ρ). Although this was an unexpected result, it provides a potentially beneficial option for practical applications. By combining PT-DCD and IPT-DCD into a hybrid system, using a threshold of ρ=0.18, the system can effectively adapt across a wide range of communication conditions—from typical delays to extreme cases. Although real-time measurement of the contamination ratio (ρ) has not been extensively studied, the method was previously discussed for estimating ρ in real time in [51]. Utilizing such real-time estimation methods to select appropriate predictors dynamically is expected to significantly improve the performance of the predictor in reducing communication delay.

### 5.4. Towards Practical Application and Extension

Although teleoperation technologies can be widely applied in various fields (e.g., surgery, mobility, and unmanned ground vehicles), the degree of performance improvement may vary across systems, requiring system-specific research. To achieve the desired performance of the predictors that mitigate communication delays, it is highly recommended to consider resampling the control target datasets. Readers should recognize that this study specifically focused on vehicle steering angle in remote driving systems, and thus the proposed method may not perform optimally under different conditions. In particular, due to the sensitivity of the model to training parameters, sufficient performance improvement may not be achieved unless the training data are collected directly from the target system. Additionally, even though high integrity in measurement is required, real-world data can often be noisy and sensitive. With the aid of synchronization tools such as chrony, this study assumed that the precise measurement of steering torque data and accurate delay computation were possible. Therefore, in order to apply the proposed predictor to real-time systems, accurate communication delay estimation and an environment capable of providing reliable measurement data are essential.

## 6. Conclusions

We proposed IPT-DCD, a deep learning-based interpolation predictor designed to mitigate dynamic communication delays in teleoperation environments. Experimental results under varying outlier contamination ratios (ρ) of communication delays demonstrated that IPT-DCD exhibited greater robustness than PT-DCD under high contamination conditions, whereas PT-DCD demonstrated better performance under passive communication delays. These findings suggest that the two methods possess complementary strengths in delay compensation: PT-DCD is more effective for passive delays, while IPT-DCD excels under delay outliers. Consequently, by dynamically selecting predictors based on ρ, delay compensation can be optimized across a wide range of real-world communication scenarios. This work lays a foundation for practical and delay-resilient teleoperation. The proposed approach can be applied not only to teleoperation systems such as microsurgery [4], mobile robots [14], and unmanned ground vehicles (UGVs) [11], but also to other domains requiring predictive compensation mechanisms, such as impedance control in human–robot collaboration [52]. Future work may explore advanced hybrid frameworks, real-time estimation of ρ, and multi-modal prediction architectures.

## Figures and Tables

**Table 1:** Applications and communication delays of recent teleoperation studies.

Application	Author(s)	Year	Delay	Achievement(s)
Surgical robot (Energy dissection and Needle task)	Song et al. [8]	2014	0∼1000 ms	Investigated effects of latency on surgical performance and its acceptable latency levels
Surgical robot (Camera targeting and Needle task)	Perez et al. [9]	2016	100∼1000 ms	Gradually increasing latency has a growing impact on performance, and measurable deterioration begins at 300 ms
Surgical robot (Block transfer)	Lum et al. [10]	2009	0, 250, 500 ms	Lab results indicated degradation of teleoperation performance as delay increased, using completion time and tool path length as metrics
Vehicle (UGVs)	Zheng et al. [11]	2020	900 ms (RTT)	Achieved higher vehicle speed, more accurate lateral control, and better drivability
Vehicle (UGVs)	Gnatzig et al. [12]	2013	121 ms (RTT)	Average RTT measured for a 3G-based teleoperated vehicle. Peak delays were >1 s
Vehicle (UGVs)	Appelqvist et al. [13]	2007	330 ms (control), 550 ms (video)	Demonstrated high-bandwidth WLAN video with low-bandwidth radio link control signal
Mobile robot	Olakanmi et al. [14]	2019	54∼251 ms	Developed a tele-autonomous model vehicle with high-performance drivetrain and efficient servo-controlled steering

**Table 2:** Summary of Related Works.

Approach	Author(s)	Method(s)	Pros	Cons
Model-based predictor	Zhang et al. [32]	Improved maneuverability under large delays	Performance improvements greater than 200 ms	Depends on the accuracy of the deep learning model
	Wang et al. [33]	Bilateral neural network controller	Accounting both linear and nonlinear models	Long computation time
	Penizzotto et al. [34]	Risk-based command metrics	Maintaining a balance between safety, transparency and completion time	Accurately assessing risk in real-time can be complex
	Chucholowski et al. [35]	Three prediction methods are proposed to improve teleoperated driving	Simplified computation and sensor requirements	Depend on exact vehicle parameters
Model-free predictor	Zhao et al. [36]	Derivative properties to compensate for overshoot	Reduce prediction error by 25%	Performance degrades as latency increases
	X. Ge et al. [37]	No need knowledge of vehicle dynamics	Easy to implement on various teleoperated systems	Additional robustness analysis needed
	Zheng et al. [38]	Allows the same predictor to be used across multiple vehicles	Reduce track completion time by 35% and track maintenance errors by 50% compared to delayed cases	The design parameter λ needs to be adjusted
	Zheng et al. [15]	Stable structure expansion	Simple implementation and robustness to modeling errors	The predictor may not be effective at high-frequency tasks

**Table 3:** Notation table in problem definition.

Notation	Description
$\tau_{\mathrm{RTT}}\left(t\right)$	A round trip communication delay at time
*i*	Each subsystem, {1,2} ∈ *i* (i.e., operator and controlled devices)
$\tau_{i}\left(t\right)$	Communication delays for each subsystem
$x_{i}\left(t\right)$	State space vectors in each subsystem
${\hat{x}}_{i}\left(t\right)$	Predicted state space vectors in each subsystem
$g_{i}\left(\cdot \right)$	Nonlinear function for prediction
$h_{i}\left(\cdot \right)$	Nonlinear function for observation
ρ	A contamination ratio of the communication delay outlier, 0 ≤ ρ ≤1
$N(\mu ,s^{2})$	Gaussian distribution for the mean and standard deviation of the delay
$\mu_{p}$, $\mu_{o}$	Mean of each passive delay and outlier delay
$s_{p}$, $s_{o}$	Standard deviation for each passive delay and outlier delay
ϵ	An error vector

**Table 4:** Notation table in LSTM.

Notation	Description
$c_{t}$	A current content vector using both the forget and input gates
$f_{t}$	A forget gate term
$i_{t}$	A input gate term
${\tilde{c}}_{t}$	A new candidate information
σ	A non-linear function
$W_{i_{h}}$,$W_{o_{h}}$	A weight matrix for input and output gates
$h_{t}$	A hidden state cell
$x_{t}$	A input value
$b_{i}$,$b_{o}$	A bias vector regarding input and output gates
$o_{t}$	A output gate

**Table 5:** Notation table in PT-DCD.

Notation	Description
$H_{p}$	A non-linear function for prediction of the PT-DCD
${\tilde{X}}_{k}$	Matrix of the input gate vector
${\tilde{Y}}_{k}$	Matrix of the output gate vector
$\tilde{\theta }$	A observed control signal
$\tau_{1}$	A control delay at discrete time
$g_{p}\left({\tilde{X}}_{k}\right){|}_{n}$	An *n*th element vector of output
$\epsilon_{p}$	An error vector for prediction

**Table 6:** Notation table in IPT-DCD.

Notation	Description
$g_{\mathrm{ip}}$	A non-linear IPT-DCD LSTM function
${\tilde{X}}_{t}$	A state space matrix at time *t*
Θ	An LSTM network parameters
${\tilde{Y}}_{t}$	An output matrix at time t
${\tilde{\theta }}_{t}$	An observed control signal at time *t*
${\tilde{X}}_{k}^{†}$	An observed state space model
$T_{s}$	An original sampling frequency of the control signal
Δt	A desired time nterval using a preliminarily determined interpolation rate
*P*	A final input state space matrix
L	A loss function
*N*	A total number of datasets

**Table 7:** Training configuration for IPT-DCD.

Layers	Hidden	Input Size	Input/Output Window	Epoch	LR	Dropout	Optimizer
2	128	3	20/10	54	0.0005	0.1	Adam

**Table 8:** Computational performances used in the training and experiment.

CPU	GPU	OS	CUDA	Python	Pytorch
i9-14900KF	RTX 4090	Ubuntu 22.04	12.4	3.10.16	2.6.0

**Table 9:** RMSE comparison between delayed and predicted steering angles using the PT-DCD.

ρ	0	0.1	0.3	0.5
Delayed (deg)	0.2098	0.5295	0.8040	0.9593
PT-DCD (deg)	0.1258	0.4004	0.6334	0.7698
RMSE reduction (%)	40.07	24.38	21.22	19.75

**Table 10:** RMSE comparison between delayed and predicted steering angles using the IPT-DCD.

ρ	0	0.1	0.3	0.5
Delayed (deg)	0.2098	0.5295	0.8040	0.9593
IPT-DCD (deg)	0.2085	0.4429	0.5839	0.6707
RMSE reduction (%)	0.6410	16.37	27.38	30.09

**Table 11:** *p*-values resulted from Shapiro–Wilk test.

ρ	0	0.1	0.3	0.5
Delayed	0.8660	0.4929	0.8125	0.02715
PT-DCD	0.4430	0.4197	0.8451	0.03032
IPT-DCD	0.5464	0.2666	0.3528	0.6841

**Table 12:** *p*-values resulted from Kruskal–Wallis H test and Dunn’s test for each ρ value.

ρ=0			
	**Delayed**	**PT-DCD**	**IPT-DCD**
Delayed	1.000×10	$1.055\times 10^{-36}$	$3.044\times 10^{-1}$
PT-DCD	$1.055\times 10^{-36}$	1.000×10	$2.173\times 10^{-31}$
IPT-DCD	$3.044\times 10^{-1}$	$2.173\times 10^{-31}$	1.000×10
ρ=0.1			
	**Delayed**	**PT-DCD**	**IPT-DCD**
Delayed	1.000×10	$8.655\times 10^{-50}$	$1.002\times 10^{-19}$
PT-DCD	$8.655\times 10^{-50}$	1.000×10	$9.187\times 10^{-9}$
IPT-DCD	$1.002\times 10^{-19}$	$9.187\times 10^{-9}$	1.000×10
ρ=0.3			
	**Delayed**	**PT-DCD**	**IPT-DCD**
Delayed	1.000×10	$1.763\times 10^{-19}$	$1.038\times 10^{-52}$
PT-DCD	$1.763\times 10^{-19}$	1.000×10	$4.135\times 10^{-10}$
IPT-DCD	$1.038\times 10^{-52}$	$4.135\times 10^{-10}$	1.000×10
ρ=0.5			
	**Delayed**	**PT-DCD**	**IPT-DCD**
Delayed	1.000×10	$6.726\times 10^{-16}$	$3.233\times 10^{-59}$
PT-DCD	$6.726\times 10^{-16}$	1.000×10	$6.726\times 10^{-16}$
IPT-DCD	$3.233\times 10^{-59}$	$6.726\times 10^{-16}$	1.000×10

**Table 13:** Cohen’s d and effect size interpretations for group comparisons at different ρ values.

ρ=0.0		
**Group Comparison**	**Cohen’s d**	**Effect Size**
1 (Delayed)—2 (PT-DCD)	16.636	Large
1 (Delayed)—3 (IPT-DCD)	0.212	Medium
2 (PT-DCD)—3 (IPT-DCD)	15.972	Large
ρ=0.1		
**Group Comparison**	**Cohen’s d**	**Effect Size**
1 (Delayed)—2 (PT-DCD)	4.021	Large
1 (Delayed)—3 (IPT-DCD)	3.011	Large
2 (PT-DCD)—3 (IPT-DCD)	1.416	Large
ρ=0.3		
**Group Comparison**	**Cohen’s d**	**Effect Size**
1 (Delayed)—2 (PT-DCD)	5.462	Large
1 (Delayed)—3 (IPT-DCD)	7.258	Large
2 (PT-DCD)—3 (IPT-DCD)	1.672	Large
ρ=0.5		
**Group Comparison**	**Cohen’s d**	**Effect Size**
1 (Delayed)—2 (PT-DCD)	7.167	Large
1 (Delayed)—3 (IPT-DCD)	11.438	Large
2 (PT-DCD)—3 (IPT-DCD)	3.952	Large

## Data Availability

https://huggingface.co/datasets/commaai/commaSteeringControl, accessed on 27 June 2025.
